# The Indications Afforded by the Pupil

**Published:** 1877-03

**Authors:** 


### The Indications Afforded by the Pupil.

Numerous and valuable as are the indications furnished by the pupil, its condition does not receive from many the attention it demands. Most of us have certain vague notions on the subject, and all are aware that the pupil is liable to alterations in certain diseases. But a systematic arrangement of its deviations from the normal standard, with explanations of their causation, is still a desideratum. The subject is one of great intricacy, for the behavior of the pupil is sometimes paridoxical, and frequently it is difficult of explanation.

The small pupil of plethoria and the dilated pupil of anæmia are constantly impressed on our notice, and appear to find simple and sufficient explanations in the fulness of the vessels and the quality of the blood. If we purge the plethoric patient, his pupils enlarge; and we estimate the anæmic patient's progress towards recovery by the diminuition in the size of his pupils. Apart from general constitutional states on which it may depend, a contracted pupil indicates cerebral hyperæmia, whilst a dilated pupil affords the most valuable sign of cerebral anæmia. Thus the small pupil of the "ferrety eye" of typhus is aid to diagnosis and a good indication of the engorgement of the encephalic centres. Again, contrast the small pupil of mitral regurgitation with the dilated pupil of aortic insufficiency. In the various affections in which pulmonary circulation is embarrassed, the small pupil attracts attention no less than the lividity of the lips and the turgescence of the cheeks. So much is myosis an indication of venous engorgement in capillary bronchitis, pulmonary œdema, and the like, that the degree of the one may be estimated by the degree of the other, and it is observed that, if the livid patient with contracted pupil be bled, the pupil dilates as the blood flows. As a rule, the condition of the pupil may be taken to indicate the state of the circulation, perhaps more in relation to fulness of the vessels than to actual blood-pressure, some of the observations, however, on this point appear contradictory and require investigation.

In connection with nervous affections, the pupil has long engaged attention, and the older physicians carefully noted its behavior in certain diseases. In the various forms of meningitis and cerebritis, where there are signs of cerebral excitement, the pupil is small. But as stupor supervenes on excitement, the pupils become oscillating and dilated; and if this passes into profound coma, they are found widely dilated and immobile. In epilepsy, too, the different stages of the paroxysm, and the condition of the circulation which accompanies or causes them, find an exponent in the state of the pupil. A transitory dilation of the pupil is amongst the most valuable signs of an attack of *petit mal.* Again, in distinguishing between feigned and real convulsive seizures, the pupil serves the physician as a valuable guide. In the appoplectic patient, pinhole pupils enable the physician to locate the hæmorrhage in the pons Varolii; whilst great dilation of one pupil occurring coincidentally with hemiplegia of the opposite side is an indication of a lesion of the crus cerebri of the same side as that on which the eye is affected.

In aneurism of the aorta, the attention of the physician is sometimes first directed to the patient's malady by a difference in the pupils. In some diseases of the spinal cord—in locomotor ataxia especially—the pupils are almost habitually contracted. In the practice of the alienist the greatest value is attached to the state of the pupil, and he is almost able, for purposes of treatment, to classify his patients by his sign alone. In toxicology it serves as an important means of distinguishing between many poisons; and oftentimes the analyst makes use of the pupil in testing for some of the alkaloids.

But not only is the pupil of great service as a clinical guide—especially as a hæmadynamometer—but it has been recently shown that in it we have an instrument of the greatest scientific precision for testing sensibility. By its employment as an æsthesiometer results have been obtained which other instruments of research have failed to reveal. Heidenhain, Meischer, Dittmar, and others, have shown that irritation of a sensory nerve is followed by a rise of blood-pressure, and have employed arterial tension in this way as a test of sensibility. But Fox and Schiff have demonstrated that in the pupil we have a test of far greater delicacy. They have shown that in the currarised animal, kept alive by artificial respiration, each irritation of a sensory nerve is followed by dilation of the pupil. This occurs not only when the effect is painful, but as the result of a simple tactile sensation. Simple pressure on the integument is sufficient to cause dilation of the pupil; and if the skin, rendered pale by pressure, be again touched, the pupil dilates still further. By this method they have ascertained that there is no portion of the body which is not possessed of feeling in this sense. In comparison with the blood-pressure, they show that dilation of the pupil follows upon excitation, before its effects can be observed in the circulation, and that a tactile sensation too feeble to evoke a rise of blood-pressure causes an alteration in the pupil. In the dilation of the pupil is afforded an indication of such sensation having reached the brain, whereas an alteration of the blood-pressure may be affected without any such conseiousness, its effects being expended on the spinal cord and medulla. The proof of this assertion is afforded by the effect of electrical excitation. If a current be applied to the anterior or lateral columns of the cord, there follows au increased blood-pressure, but no dilation of the pupil. The reflection from sensory nerves to motor nerves takes place in the cord, from censory nerves to the pupil of the brain. In accordance with these facts the condition ot the pupil has been proposed by Budin and Coyne as a guide in chloroform narcosis, but in this condition it affords neither safe nor satisfactory indications.—*Med. Exam.*

